# 

**Published:** 1986-02

**Authors:** 


					VOLUME 101 (i)
FEBRUARY 1986
Chronic Venous Ulcers of the Leg
"Skins are simpler than you think"
Starting to Sail
Current Comment from Correspondents
IN ASSOCIATION WITH
BRISTOL MEDICINE
THE MEDICAL JOURNAL OF THE SOUTH WEST

				

